# Preliminary assessment of a tool for measuring relationship-centred communication in veterinary consultations (adapted VR-COPE)

**DOI:** 10.15694/mep.2021.000134.1

**Published:** 2021-05-17

**Authors:** Michael McDermott, Malcolm Cobb, Iain Robbé, Rachel Dean

**Affiliations:** 1 Circa Healthcare; 2 University of Nottingham School of Veterinary Medicine and Science; 3 The Royal (Dick) School of Veterinary Studies; 4 VetPartners Ltd.

**Keywords:** Veterinary communication, veterinary consultations, relationship-centredness, VR-COPE, VR-CoDES

## Abstract

This article was migrated. The article was marked as recommended.

Relationship-centred communication is considered a desirable goal in veterinary medicine, and a number of different tools have been developed to measure relationship-centred communication. This study was designed as an initial assessment of an adapted version of the Verona Patient-centred Communication Evaluation (VR-COPE) scale, originally developed for medical communication research, to evaluate its potential for measuring relationship-centredness in veterinary consultations. Fifty-five consultations in the United Kingdom and United States of America were videotaped and analysed. The median VR-COPE total score (out of a potential 100 points) was 76.00 for all consultations. The highest overall score was for “Structuring” (of the consultation), whereas the lowest scores were for “Client Worries,” “Psychological Impact,” and “Empathy.” This initial assessment of the adapted VR-COPE suggests it may be helpful in measuring content, process, and structuring skills related to relationship-centredness in veterinary consultations. It may also help uncover aspects of relationship-centredness that are unique from those uncovered by other tools. Further research is needed to fully assess the role of VR-COPE in veterinary communication research and the contributions it can make to relationship-centredness in veterinary consultations.

## Introduction

Relationship-centred communication in human medicine involves a sharing of dialogue and decision-making between a healthcare professional and patient ( Beach and Inui, 2006). In veterinary medicine, this term has been used to describe communication during a consultation involving a veterinary professional, client and patient, ( Cornell and Kopcha, 2007; Hamood, Chur-Hansen and McArthur, 2014; Vijfhuizen *et al.,* 2017), which is triadic in nature and in which the patient does not speak, similar to a paediatric consultation with a non-verbal patient ( Keir and Wilkinson, 2013; Tates and Meeuwesen, 2001). This adds an extra level to the veterinary consultation that can influence the dynamic, considering that the veterinarian, client, or patient can be the focus of communication delivered by the first two parties of the triad ( Hamood, Chur-Hansen and McArthur, 2014; Vijfhuizen *et al.,* 2017).

Relationship-centred communication is considered a desirable goal in veterinary communication, and is characterised by the use of open questions, active listening, making eye contact, including the client’s thoughts and opinions, and demonstrating empathy and understanding of the client’s perspective ( Cornell and Kopcha, 2007; Abood, 2007; Shaw, 2006). In recent years relationship-centred communication has gained increasing importance in veterinary communication training in veterinary school and in continuing professional development (CPD) ( Burns *et al.,* 2015; Mossop and Gray, 2008; Mossop *et al.,* 2015; Shaw and Ihle, 2006).

A number of different tools have been used to assess relationship-centred communication. In most cases, the tool was originally developed for assessment of medical professional-patient communication in human medicine then adapted for veterinary communication research. One example is The Roter Interaction Analysis System (RIAS) ( Shaw *et al.,* 2004; Roter and Larson, 2002; Shaw *et al.,* 2016; Borden *et al.,* 2010). RIAS has been the most commonly used tool for measuring relationship-centredness in both medical and veterinary communication research ( Shaw *et al.,* 2004; Shaw *et al.,* 2010; Shaw *et al.,* 2016; McCarthy *et al.,* 2013). It characterises dialogue during a consultation by using discrete codes to characterise each complete thought in the conversation, using an audio or video recording of the consultation to conduct the analysis ( Roter and Larson, 2002). The RIAS codes are organised into task-focused categories (data gathering, patient education and counselling), and socio-emotional exchange categories (building a relationship, patient activation), each with corresponding communication behaviour, for example asking open- versus closed questions about medical or psychosocial topics under “data-gathering,” or social talk under “building a relationship.” A relationship-centredness score is determined by the ratio of dialogue segments coded for psychosocial and socio-emotional topics to those coded for biomedical topics ( Roter and Larson, 2002).

The Verona Patient-centred Communication Evaluation (VR-COPE) scale ( Del Piccolo *et al.,* 2008) was developed by Lidia del Piccolo at the University of Verona to evaluate both content and relational elements of medical consultations across nine separate criteria related to relationship-centredness ( Del Piccolo *et al.,* 2008). The nine categories and sub-categories are related to Content (what the physician and patient talk about), Skills (the demonstration of the healthcare provider of verbal or nonverbal communication skills associated with relationship-centred communication) and/or Process (how the physician structures the consultation according to accepted best practices) ( Del Piccolo *et al.,* 2008). A score from 0 to 10 is given for each of the nine categories, based on a review of the dialogue during the consultation, with 90 representing the highest possible degree of relationship-centredness ( Del Piccolo *et al.,* 2008).

The VR-COPE has been used in studies of medical consultations, beginning with the initial development of the tool, for which nearly 250 transcribed medical consultations were rated across the nine variables. In a recent study of newly diagnosed breast cancer patients ( Del Piccolo *et al.,* 2019), the VR-COPE was used to assess relationship-centredness of oncologists in initial consultations. VR-COPE scores were highest for elements of the tool related to structuring the consultation and gathering information from, and providing information to, the patient and lowest in evaluating the emotional impact of the disease on the patient, responding to patient worries and emotional needs, and inquiring about the patient’s expectations or point of view ( Del Piccolo *et al.,* 2019). Until the present study, the VR-COPE had not been used in veterinary communication research.

Another tool developed by Del Piccolo and colleagues is the Verona Coding Definitions of Emotional Sequences (VR-CoDES) ( Zimmermann *et al.,* 2011), which is used to record patient expressions of negative emotions in the form of cues or concerns. Cues are implicit expressions of potential emotional concerns whereas concerns are explicit expressions where the concern is clearly defined. VR-CoDES has been used in communication research in human and, more recently, veterinary medicine ( Zimmermann *et al.,* 2011; Del Piccolo *et al.,* 2019; Del Piccolo *et al.,* 2015; Vijfhuizen *et al.,* 2017).

The VR-CoDES were adapted by Vijfhuizen *et al.* (2017) for use in veterinary research and used to analyse 20 veterinary consultations for emotional cues and concerns. As has been noted in medical communication research ( Zimmermann *et al.,* 2011) cues were more frequently noted than concerns, with cues being noted in all 20 of the consultations and concerns only in 10. The most common veterinarian response to a client’s cue or concern was ignoring it, which happened in more than 30% of cases. When responses occurred, they were often non-explicit or factually acknowledged the client’s expression without acknowledging the emotional element of it ( Vijfhuizen *et al.,* 2017). This lack of attention to emotions and concerns was consistent with previous studies in veterinary communication ( Butler, Williams and Koll, 2002; Shaw *et al.,* 2008; McDermott *et al.,* 2020).

The aim of this study was to conduct a preliminary assessment of the VR-COPE for its potential to be considered as an additional tool to assess relationship-centredness in veterinary consults in ways that are different from the information available from other tools.

## Methods

### Study Practices

The study was conducted in five veterinary practices in the United Kingdom (UK), comprised of three first opinion practices, one referral practice, and one combination first-opinion and referral practice, from 20 October 2014 through 24 October 2014, and in five first-opinion practices in the United States of America (USA), from 24 November 2014 through 22 June 2015.

### Practice and veterinary surgeon recruitment

A convenience sample of ten practices was selected among contacts within a practical proximity to the Centre for Evidence-based Veterinary Medicine (CEVM) in the UK and the primary author’s (MMcD) home base in the USA. Practice owners were contacted in person or by phone and were provided with a written description of the study as well as a consent form, which each veterinary surgeon signed before or upon arrival at the practice on the day of data collection.

### Client recruitment

On arrival at the clinic prior to their appointment, clients were asked if they were willing to participate in the study. A written and verbal explanation of the focus and purpose of the study were provided. These included acknowledgement that the consultation would be video-recorded and there would be a post-consultation survey. All participants were required to sign consent forms prior to their consultations.

### Video-recording

The video-recording methodology was informed by a previous study done by Everitt *et al.* (2013). For each of the practices, MMcD (and for UK practices, a colleague from the research institution) arrived before the first consultation to be recorded on each day. Cameras (2) were situated in the examination room to capture as much of the consultation action and dialogue as possible and allowed to run for the length of each consultation. After each day of video-recording, the digital video recordings of the consultations were uploaded to MMcD’s laptop computer, with file names indicating country, practice, and consultation number.

### Adaptation of the study tool

**Figure 1:**
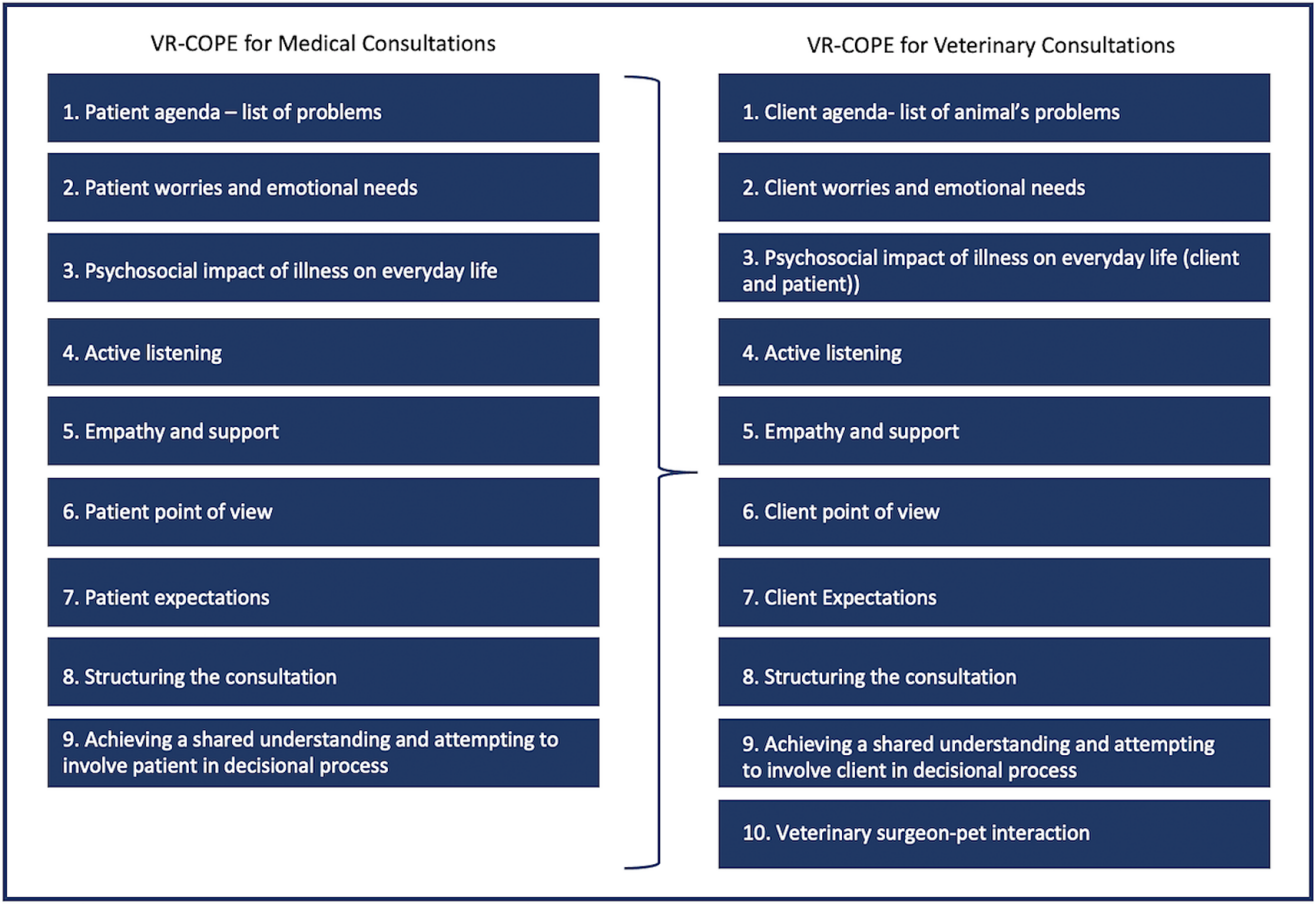
Original VR-COPE for Medical Consultations and Adapted VR-COPE for Veterinary Consultations

In order to optimise the utility of the VR-COPE for veterinary consultation, the authors adapted the tool in three stages. In the first stage, the tool was reviewed, and some wording was changed to more closely reflect the participants and activities of a veterinary consultation (e.g., client instead of patient, veterinary surgeon instead of physician, effect of client’s illness on everyday life instead of effect of patient’s illness, etc.). A tenth element was added, “Veterinary Surgeon-Pet Interaction,” to reflect the triadic nature of the veterinary consultation. This element referred to the veterinary professional speaking to or physically engaging (e.g., touching or stroking) the animal, not including physical examinations. In the second stage, two postgraduate student colleagues analysed two pilot consultations using the tool adapted in stage one. The colleague feedback on their experience with the tool resulted in further recommendations for the adaptation of the tool. The third stage was the creation of the final adapted version of the VR-COPE for veterinary research (see Figure 1).

### Consultation length

Length of each consultation was recorded from the video timer and transferred to an Excel spreadsheet for further analysis.

### Consultation review and scoring

Using a process agreed by all authors, MMcD viewed each of the video-recorded consultations from both cameras and using a form that contained all the elements of the adapted VR-COPE and their descriptions, manually entered a score from 1 to 10 for each element. The total adapted VR-COPE score was the sum of all 10 element scores (maximum 100). Once this step was completed for all practices, the data were reviewed and transferred to an Excel spreadsheet for analysis.

### Consultation and adapted VR-COPE data analysis

Descriptive statistics for the consultations and adapted VR-COPE analysis were generated by importing the data from the Excel spreadsheet into SPSS ^®^ (IBM Corp., Relased 2015. IBM SPSS Statistics for Windows, Version 23.0, Armonk, NY: IBM Corp.) and calculating median, range, and interquartile range (IQR) for the consultation time and score assigned to each adapted VR-COPE element as well as the total adapted VR-COPE score for all consultations, all UK consultations, and all USA consultations. For each element of the client-centredness analysis, the median scores for UK and USA consultations were compared using Mann-Whitney U Tests (significance at p<0.05).

## Results/Analysis

### Consultations

Fifty-five consultations were analysed, 28 in the UK and 27 in the USA. Median length of consultation overall was 16.78 minutes for all consultations, 16.06 minutes for UK consultations, and 16.78 minutes for USA consultations, with no significant difference between length of UK and USA consultations. Number, date, and length of consultations appear in Table 1.

**Table 1:** Number and Length of Consultations

Country	Date	Practice Code	Consultations/ Practice	Consultation Length (Minutes)			Comparison UK to USA (Length of Consultation)
				Median	Range	IQR	
**All Practices**			55	16.78	5.25-44.18	11.36, 20.28	
**UK Practices**			28	16.06	5.25-44.18	9.52,18.98	Not significantly different(p=0.167)
**UK**	20 Oct 2014	UK1	6	9.94	8.32-30.42	8.81,18.64	
	21 Oct 2014	UK2	6	16.86	9.52-18.98	11.79,18.38	
	22 Oct 2014	UK3	5	16.06	8.25-19.05	10.25,17.96	
	23 Oct 2014	UK4	4	21.35	5.65-23.62	9.23,23.40	
	24 Oct 2014	UK5	7	17.90	5.25-44.18	14.35,22.07	
**USA Practices**			27	16.78	7.77-29.57	11.92,21.87	
**USA**	24 Nov 2014	US1	5	18.86	13.28-25.83	15.92,23.06	
	02 Feb 2015	US2	5	15.92	8.41-23.24	11.42,22.56	
	23 Feb 2015	US3	5	19.20	8.80-28.02	11.89,25.80	
	20 Apr 2015	US4	6	16.98	7.77-29.57	10.46,28.73	
	22 Jun 2015	US5	6	13.97	9.05-18.61	9.72,17.24	

### Adapted VR-COPE total score

**Figure 2:**
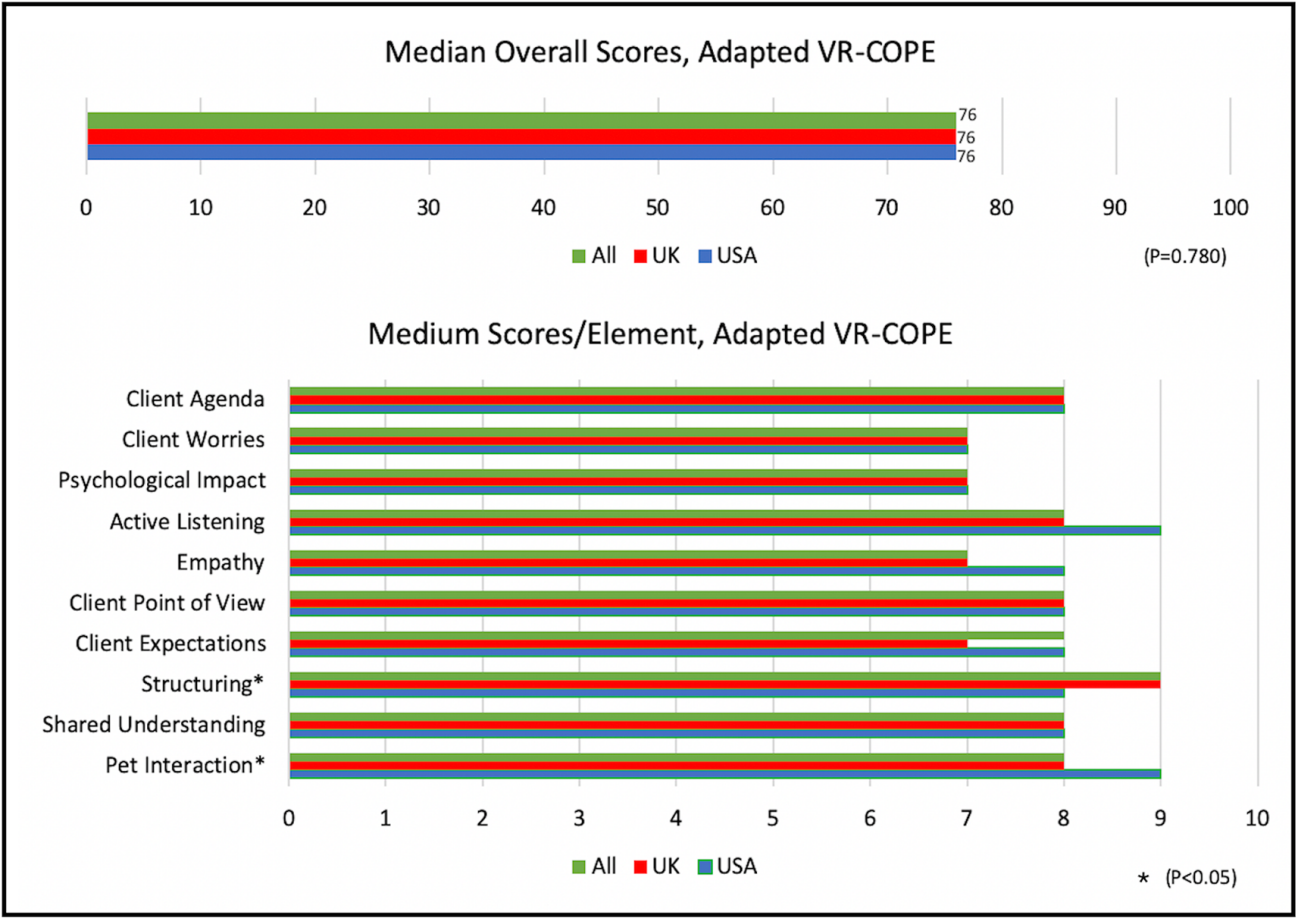
Median Overall Scores and Scores for Elements, Adapted VR-COPE

The median VR-COPE total score (out of a potential 100 points, with 100 representing the highest degree of relationship-centredness) was 76.00 for all consultations, UK consultations, and USA consultations, with no significant difference between the UK and USA (P=0.780). These results appear in Figure 2.

### Adapted VR-COPE high and low element scores

The adapted VR-COPE element reported with the highest overall score was “Structuring” (of the consultation) (median 9, range 6-10, IQR 8,9). The adapted VR-COPE elements reported with the lowest overall scores were “Client Worries,” (median 7, range 5-9, IQR 6,7), “Psychological Impact,” (median 7, range 4-10, IQR 6,8), and “Empathy” (median 7, range 5-9, IQR 6,8). Overall scores (combined, UK practices, and USA practices) for each of the 10 elements appear in Figure 2.

### Adapted VR-COPE elements with significant differences between the UK and USA

The score for “Structuring the Consultation” was significantly higher in the UK (median 9, range 8-10, IQR 8,9) than in the USA (median 8, range 6-10, IQR 8,9); (P=0.041). The score for “Veterinary Surgeon-Pet Interaction” was significantly higher in the USA (median 9, range 6-10, IQR 7,10) than in the UK (median 8, range 5-10, IQR 6,9); (P=0.024). There were no other significant differences in overall scores for adapted VR-COPE elements between the US and USA.

## Discussion

This initial experience with the VR-COPE adapted for veterinary consultations demonstrated the utility of the tool for measuring content, process, and structuring skills related to relationship-centredness in veterinary consultations. Because of its inclusion of these three elements of a veterinary consultation, VR-COPE is able to uncover aspects of relationship-centredness that are unique from those uncovered by RIAS or VR-CoDES. This is not to say that VR-COPE is a better analysis tool than the others, rather that VR-COPE, in combination with RIAS and VR-CoDES, could potentially provide additional useful insights into the relationship-centredness of a veterinary consultation. More studies would be required to determine the extent and value of these additional insights.

It was possible to use the adapted VR-COPE to explore elements of client-centred communication that have been demonstrated as important promoters of client understanding and client satisfaction, including soliciting client feelings, concerns, and input, and expressing empathy and understanding in discussions with clients about the health of their pets ( Abood, 2007; Coe *et al.,* 2010; Mellanby, 2011; Shaw *et al.,* 2012). The addition of the “Veterinary Surgeon-Pet Interaction” component was useful, as there were several examples of a veterinary professional verbally or physically engaging the pet during the consultations. Some aspects of the veterinary surgeon’s acknowledging and responding to client concerns, worries, and emotions were less easy to assess with the adapted VR-COPE, because these were not often expressed strongly in the studied consultations.

Scores for components related to client emotions and the veterinary surgeon responding to them were generally lower than those related to structuring the consultation and gathering information, which is consistent with the dominance of biomedical over psychosocial dialogue as well as the lesser attention to emotion seen in other veterinary communication studies ( Shaw *et al.,* 2004; Shaw *et al.,* 2008; Shaw *et al.,* 2006; Vijfhuizen *et al.,* 2017) and in a study of breast cancer patients in which the original version of the VR-COPE was used ( Del Piccolo *et al.,* 2019).

Differences in scores of UK versus USA consultations for “Structuring the Consultation” (higher in the UK) and “Veterinary Surgeon-Pet Interaction” (higher in the USA) might reflect different emphases placed on these activities in the two countries, but the number of consultations studied and geographic concentration of the two sets of practices were too small to draw any broad-based conclusions about differences in the way consultations are conducted in each country. Further research would be required to determine if the results were specific to this study or indicative of consultations in the countries in general.

In order to more fully assess the utility of the VR-COPE for veterinary communication research, it is recommended that additional studies be conducted on larger numbers of consultations. The VR-COPE could be further adapted for veterinary consultations to more accurately characterise the Content, Process, and Structuring skills of a relationship-centred veterinary consultation. Using the adapted VR-COPE in studies of a wider variety of patient cases, including those involving more serious illness or injury, might better test the ability of the tool to measure expression of empathy or response to emotional concerns.

Analysing a suitably sized sample of veterinary consultations with the adapted VR-COPE, the adapted VR-CoDES, and RIAS could help further determine the ability of the adapted VR-COPE to contribute novel insights to veterinary communication research. A study such as this would facilitate the assessment of client centredness from three different but potentially complementary angles: biomedical versus psychosocial dominance in dialogue, support of relationship-centeredness through content, process, and structuring, and expression and reaction to negative emotional cues and concerns.

### Limitations

The VR-COPE has never been employed in veterinary research, and therefore it was left to the authors to determine how it should be adapted for this study. Though a pilot study was conducted in which two colleagues independently rated consultations and these scores were used in the refinement of the tool in the iterative adaptation, where was no formal inter-rater reliability test done on the pilot. Even though the current study was not designed to be a formal validation of the VR-COPE for veterinary research (the original validation of the VR-COPE was done with 246 consultations), an IRR calculation could have provided initial evidence of the utility of the tool and might have facilitated additional adaptation. The sample size of 55 consultations was also relatively small, another reason why the current study should be considered a preliminary assessment of the tool and not a definitive validation.

## Conclusion

This initial evaluation of the adapted VR-COPE in veterinary research demonstrated that it can assess unique and valuable aspects of client-centredness in veterinary surgeon-client communication. Further development of the VR-COPE, along with its use in studies of greater sample size, on a wider variety of cases, and perhaps in combination with assessment by RIAS and/or VR-CoDES, could help to more firmly determine the place of this unique tool in veterinary communication research.

## Take Home Messages

• Relationship-centred communication is recognised as a desired objective in a medical or veterinary consultation
• The VR-COPE, a tool developed for developing relationship centredness in medical communication research was adapted for use in studying veterinary consultations, and piloted in 55 veterinary consultations in the United Kingdom and in the United States of America
• In this study, the highest overall score was for “Structuring” (of the consultation), whereas the lowest scores were for “Client Worries,” “Psychological Impact,” and “Empathy,” which is consistent with previous research in medical and veterinary communication research
• The VR-COPE may add new information for the understanding of veterinary surgeon-client-patient communication in that it may be used to assess relationship-centredness from the perspectives of Content, Skills, and Process
• Further research is needed to fully assess the role of VR-COPE in veterinary communication research and the contributions it can make to relationship-centredness in veterinary consultations

## Notes On Contributors

**Dr. Michael McDermott** earned a PhD from the University of Nottingham School of Veterinary Medicine and Sciences in 2018, with a research focus on veterinary communication skills and training. His career in marketing communications and marketing includes roles with marketing communications agencies and with pharmaceutical and animal health companies.

**Dr. Malcolm Cobb** holds a Foundation Chair and Deputy Head of School in the University of Nottingham School of Veterinary Medicine and Science. He is Professor of Comparative Veterinary Medicine, teaching final year students and doing clinical work at Clinical Associate practices.

**Dr. Iain Robbé** is a medical practitioner (MB, BS, 1980; MRCS, LRCP, 1980) and Clinical Medical Educationist contributing to projects in both medical and veterinary schools, including the Royal (Dick) School of Veterinary Studies, University of Edinburgh. He has participated in research in both medical and veterinary communication.

**Dr. Rachel Dean** was founding Director of the Centre for Evidence-based Veterinary Medicine, and a clinical associate professor in feline medicine at the University of Nottingham. She is currently Director of Clinical Research and Excellence in Practice at VetPartners, a large group of veterinary practices in the UK.
