# Supplementary material for: Preliminary assessment of a tool for measuring relationship-centred communication in veterinary consultations (adapted VR-COPE)
Source: MedEdPublish (2016). 2021 May 17;10:134. [Version 1] doi: 10.15694/mep.2021.000134.1 (PMC10939548; doi:10.15694/mep.2021.000134.1)
Supplement: Peer review history

# Reviewer report: Reviewer response for version 1

**DOI:** 10.21956/mep.18941.r27098

**Published:** 2021-07-12

**Authors:** Trevor Gibbs

**Affiliations:** 1 AMEE

This review has been migrated. The reviewer awarded 4 stars out of 5 Not knowing that much about Vet Medicine, but as a previously practicing General Practitioner, I felt that I knew a fair amount regarding human communication , but little about veterinary communication and its assessment. I was fascinated to read this article. I must admit that I had to read it several times before fully understanding it, but I did learn much from the article and could understand where the authors were placing their research. As they say, there is a lot of similarity with paediatric care.I thought that the paper was well structured and well-presented with an appropriate conclusion from the findings. I was left a little wanting - I would have liked to have watched the videos- but that wasn't possible. However, my opinion is that it would be an appropriate paper for anyone involved in relationship -centred communication- in vet medicine or otherwise

Reviewer Expertise:

NA

No decision status is available

# Reviewer report: Reviewer response for version 1

**DOI:** 10.21956/mep.18941.r27099

**Published:** 2021-05-23

**Authors:** Carol Gray

This review has been migrated. The reviewer awarded 4 stars out of 5 The topic of the study is veterinary communication, appropriate to veterinary education. However, there are also parallels with communication in paediatric healthcare. This study offers a useful addition to the literature on evaluation of communication in both contexts, at undergraduate and postgraduate levels. It should appeal to those seeking a novel tool for quantitative assessment of veterinary professional-client communication. It provides an objective measure of the content, process and structure of veterinary healthcare consultations, and with some more work, could be adapted for clinical assessments in veterinary education at both undergraduate and postgraduate levels.The abstract provides a clear and accurate description of the study performed.The introduction sets the scene for the article, examining relationship-centred care in both human and veterinary medical settings. The authors could have taken a little more time to explain the differences in relationship-centred care in dyadic and triadic medical consultations, for example, in terms of who makes decisions about care.Limiting the tools reviewed to those utilised for quantitative analysis of communication perhaps also detracts from the introduction. A short summary of qualitative techniques (for example, conversation analysis) that provide an alternative approach may have enhanced this section.The goals of the study are clearly explained. It would have been interesting to know why the VR-CoDES was not considered either as an additional tool or as a comparator with the VR-COPE in terms of element 2 (client worries and emotional needs), especially in view of the statement in the discussion that “some aspects of the veterinary surgeon’s acknowledging and responding to client concerns, worries and emotions were less easy to assess with the adapted VR-COPE…” – would use of the VR-CoDES have facilitated this assessment?Element 3 is correct in Figure 1, but the explanation in the text is muddled (“effect of client’s illness on everyday life” should be “effect of illness on everyday life (client and patient)”).The methods are described in detail, with the use of non-intrusive remote video cameras. I would have appreciated some commentary on how remote recording may have impacted the capture of the whole context of the consultation, or how small details may have been missed by being limited to views from two cameras. A single researcher was involved in coding the consultations; however, this point is addressed adequately in the ‘limitations’ section of the discussion.Table 1 presents detailed information on the length of consultations recorded, but there is no reference to this in the discussion. I would like to see some analysis of the effect of consultation length on relationship-centred communication, and how the lengths of the consultations recorded may have impacted on this. Was there any correlation between individual consultation length and VR-COPE score? Overall, the discussion is limited to how well the VR-COPE tool performed, introducing a promising tool for analysis of veterinary consultations. As the authors suggest, it requires validation and more widespread testing, but its potential has been recognised.

Reviewer Expertise:

NA

No decision status is available
